# Midterm Outcomes for Endovascular Repair of Thoraco-Abdominal Aortic Aneurysms

**DOI:** 10.1016/j.ejvsvf.2022.03.007

**Published:** 2022-04-09

**Authors:** Håvard Ulsaker, Arne Seternes, Reidar Brekken, Frode Manstad-Hulaas

**Affiliations:** a Department of Circulation and Medical Imaging, Faculty of Medicine and Health Sciences, Norwegian University of Science and Technology (NTNU), Trondheim, Norway; b Norwegian National Advisory Unit on Ultrasound and Image-Guided Therapy, St. Olavs Hospital, Trondheim, Norway; c Department of Surgery, St. Olavs Hospital, Trondheim, Norway; d Department of Health Research – Medical Technology, SINTEF, Trondheim, Norway; e Department of Radiology, St. Olavs Hospital, Trondheim, Norway

**Keywords:** Aortic disease, Endovascular therapy, Follow up, Spinal cord ischaemia, Thoraco-abdominal aortic aneurysm

## Abstract

**Objective:**

To investigate technical and clinical outcomes in patients with thoraco-abdominal aortic aneurysms treated with the multibranched off the shelf Zenith t-Branch stent graft or a custom made device (CMD).

**Methods:**

A retrospective study was conducted of patients operated on at a single tertiary vascular centre in Norway. Twenty eight t-Branch and 17 CMD patients were identified. Demographic, aneurysm, and peri-operative data were summarised and compared.

**Results:**

Thirty day mortality was 4% (2/45), with mortality rates of 7% (2/28) and 0 in t-Branch and CMD patients, respectively (*p* = .52). Technical success was 87% (39/45), with a non-significant difference between t-Branch and CMD procedures of 89% (25/28) and 82% (14/17), respectively (*p* = .63). Stent graft coverage was significantly longer in t-Branch patients (*p* = .020). Paraparesis or paraplegia developed in 18% (5/28) of t-Branch patients and 12% (2/17) of CMD patients (*p* = .69), and spinal cord ischaemia was associated with Crawford type II aneurysms (*p* = .010) and aortic coverage >400 mm (*p* = .050). The estimated survival at one and two years for t-Branch patients was 93% and 88%, and 100% and 92% for CMD patients. Freedom from re-intervention was estimated at 70% and 43% at one and two years for t-Branch patients, and 58% and 50% for CMD patients.

**Conclusion:**

The study showed low 30 day mortality rates, acceptable technical success rates, high medium term survival, and no statistically significant differences in clinically relevant outcomes between t-Branch and CMD patients.

## Highlights

• Clinical outcomes do not vary significantly between t-Branch and CMD patients.
• 30 day mortality was low among both t-Branch and CMD patients.
• Midterm survival was high in both groups.

## Introduction

For endovascular treatment of thoraco-abdominal aortic aneurysms (TAAAs), custom made devices (CMDs) or “off the shelf” multibranched stent grafts, such as Zenith t-Branch (Cook Medical, Bloomington, IN, USA), are common treatment options. With its four branched design, the t-Branch is anatomically compatible in 60%–70% of TAAA patients.^1^ CMDs have the benefit of suiting patients’ individual aortic and renovisceral vessel anatomy and are convenient in patients with complicated thoraco-abdominal aortic configurations. CMDs normally have a production time of five to seven weeks, a period associated with a certain risk of aneurysm rupture,^2^ and are not an option when urgent repair is required.

Several retrospective studies have investigated short and medium term outcomes of endovascular TAAA repair. Key outcome measures include mortality rate, technical success, and spinal cord ischaemia (SCI). Thirty day mortality rates are reported at 10% to 0, and technical success rates are close to 100%. SCI rates vary between cohorts, and generally the literature agrees that endovascular repair for TAAAs is feasible and safe.^3^, ^4^, ^5^, ^6^, ^7^, ^8^, ^9^, ^10^, ^11^, ^12^ Follow up series are published increasingly, but there is room to further extend the knowledge base.^12^ A direct comparison of t-Branch and CMD patients was described by Bisdas and colleagues in 2014, in which the t-Branch showed comparable clinical outcomes to the more traditional CMDs.^5^

The study aim was to investigate technical and clinical outcomes in patients treated with t-Branch and CMDs and compare key endpoints.

## Methods and materials

### Design

Patients operated with t-Branch or CMD for TAAAs at St. Olavs Hospital, Trondheim University Hospital were identified using the Norwegian Vascular Surgery Registry, 45 patients in total, 28 t-Branches and 17 CMDs. Complementary data were extracted from electronic medical records. From 2014 to 2018 anatomically suitable patients were operated on using t-Branch, while from 2019 CMDs were preferred in non-urgent cases. Pre-operative computer tomography angiography (CTA) images were used to classify aneurysm extents according to Crawford and to measure aortic diameters. Follow up CTA images were used to measure post-operative aneurysm diameters and the length of aorta covered by the stent graft using Aquarius iNtuition (TeraRecon Inc., Durham, NC, USA). Aneurysm shrinkage or expansion was identified by a diameter change of ≥5 mm.

The Regional Committee for Medical and Health Research Ethics waived approval of the study. The Norwegian Centre for Research Data acted as data protection officer.

### Procedure

For t-Branch, surgical accesses were obtained bilaterally in the common femoral arteries and the right subclavian artery. Since 2019, procedures have predominantly been performed with femoral access only. The t-Branch patient selection and deployment followed the Instruction for Use manual, as described extensively elsewhere.^13^ All operations were performed single staged, except for procedures preceded by subclavian carotid bypass.

Similar set ups were used for CMD implantations, but thoracic stent graft components were seldom required. Different combinations of branches and fenestrations were used for target vessel repair.

Mean intra-arterial pressure (MAP) > 80 mmHg, haemoglobin levels >10 g/dL, and oxygen saturation 100% were aimed for in all procedures. Cerebrospinal fluid (CSF) was drained peri-operatively to <10 mmHg. CSF pressure was reduced to 5 mmHg in patients with neurological leg symptoms, and drainage was terminated 72 hours post-operatively when there were no signs of SCI.

Patients were followed up at the vascular surgery outpatient clinic with CTA imaging at six, 12, and 18 months, and thereafter annually.

### Definitions

The primary endpoint was patient survival. Secondary endpoints were technical success, SCI, and freedom from re-intervention. Technical success was defined as successful endograft deployment with bridging of all target vessels, with aneurysm exclusion without intra-operative mortality, conversion to open surgery, or persistent type 1 or 3 endoleaks.^14^ SCI was classified into either paraplegia or paraparesis.^15^

### Statistics

Continuous data are given as median (interquartile range). Categorical data are presented as counts (percentages). The Mann–Whitney *U* test and Fisher's exact test were used for between group comparisons, the Wilcoxon signed rank test for within group analyses, and the Kruskal–Wallis test for comparing length of stent graft covered aorta between Crawford classes. Time to event analyses were performed with Kaplan–Meier statistics. A *p* value ≤.050 was considered statistically significant. Analyses were performed using SPSS Statistics version 26.0.0.1 (SPSS Inc., Chicago, IL, USA).

## Results

### Demographics and thoraco-abdominal aortic aneurysm morphology

The median age was 69 years (66, 73) for t-Branch patients and 72 years (68, 75) for CMD patients (*p* = .19). The median TAAA diameter was 65 mm (61, 72) and 63 mm (59, 70) for t-Branch and CMD patients, respectively (*p* = .29). Five t-Branch patients were admitted with symptomatic TAAAs and operated on within 48 hours, one with contained rupture and four with impending rupture. Demographic and aneurysm data are summarised in Table 1.

**Table 1:** Demographics and comorbidities of patients with thoraco-abdominal aortic aneurysms treated with the multibranched off the shelf Zenith t-Branch stent graft or a custom made device (CMD)

Variables	t-Branch (*n* = 28)	CMD (*n* = 17)	*p* value
Age – y	69.0 (66.0, 72.8)	72.0 (67.5, 74.5)	.19
Male	17 (61)	11 (65)	1.00
Body mass index – kg/m^2^	26.9 (23.4, 29.3)∗	29.1 (23.9, 31.5)†	.29
Active smoker	7 (25)	6 (35)	.51
Ex-smoker	18 (64)	8 (47)	.010
Systolic BP, at admission – mmHg	134 (125, 151)	144 (138, 146)	.22
Diastolic BP, at admission – mmHg	81 (73, 88)	80 (77, 87)	.86
*Comorbidities*			
Hypertension	20 (71)	9 (53)	.34
Heart failure	2 (7)	0	.52
Coronary artery disease	7 (25)	3 (18)	.72
Peripheral artery disease	7 (25)	5 (29)	.74
Cerebrovascular disease	3 (11)	1 (6)	1.00
COPD	7 (25)	6 (35)	.51
Diabetes	2 (7)	1 (6)	1.00
*Previous aortic and cardiac surgery*			
AAA–open	9 (32)	6 (35)	1.00
AAA–EVAR	0	1 (6)	.38
Ascending/type A-dissection–open	9 (32)	2 (12)	.17
Descending/type B-dissection–open	1 (4)	2 (12)	.55
Descending/type B-dissection–TEVAR	4 (14)	3 (18)	1.00
CABG	2 (7)	2 (12)	.63
Aortic valve replacement	1 (4)	1 (6)	1.00
PCI	3 (11)	2 (12)	1.00
*Medical treatment*			
Aspirin	20 (71)	10 (59)	.052
Statins	19 (68)	12 (71)	1.00
β-blockers	14 (50)	7 (41)	.76
Warfarin	4 (14)	2 (12)	1.00
DOAC	2 (7)	1 (6)	1.00
*ASA class*			
III	19 (68)	13 (76)	.74
IV	9 (32)	4 (24)	.74
eGFR – mL/min/1.73m^2^	66.0 (49.0, 84.5)‡	62.5 (43.5, 76.3)§	.61
Creatinine – μmol/L	85.0 (78.0, 106.0)	97.0 (76.8, 116.8)§	.63
*Crawford class*			
I	3 (11)	3 (18)	.66
II	8 (29)	4 (24)	1.00
III	8 (29)	4 (24)	1.00
IV	9 (32)	5 (29)	1.00
V	0	1 (6)	.38

Data are presented as n (%) or median (IQR). BP = blood pressure; COPD = chronic obstructive pulmonary disease; AAA = abdominal aortic aneurysm; EVAR = endovascular aortic repair; TEVAR = thoracic endovascular aortic repair; CABG = coronary artery bypass grafting; PCI = percutaneous coronary intervention; DOAC = direct oral anticoagulants; ASA = American Society of Anaesthetists; eGFR = estimated glomerular filtration rate.

∗ *n* = 25 because of missing height data.

† *n* = 13 because of missing height data.

‡ *n* = 20 because of incomplete data.

§ *n* = 14 because of incomplete data.

### Peri-operative outcomes

Technical success was 87% (39/45), with 89% (*n* = 25) of t-Branch and 82% (*n* = 14) of CMD procedures being technically successful (*p* = .66). There was no difference between patients operated early and late in the period (*p* = .414). Unsuccessful procedures are detailed in Table 2.

**Table 2:** Technically unsuccessful procedures in patients with thoraco-abdominal aortic aneurysms treated with the multibranched off the shelf Zenith t-Branch stent graft or a custom made device (CMD)

Device	Technical failure
t-Branch	Emergency laparotomy as a result of bleeding from the LRA
t-Branch	LRA dissection subsequently occluded with an Amplatzer plug
t-Branch	Type 1c EL from the LRA at the final angiogram that did not resolve within 30 days
CMD	The CT branch was used for RRA stenting, and the RRA branch occluded with an Amplatzer plug
CMD	Type 3a EL by misplacement of the left iliac stent graft outside the main body inverted limb
CMD	Type 1a EL in the thoracic aorta caused by bad proximal ceiling

LRA = left renal artery; EL = endoleak; CT = coeliac trunk; RRA = right renal artery.

Of 100 t-Branch target vessels, 98 (98%) were successfully stented. For CMD vessels, 51 of 53 (96%) were successfully stented.

The median operation time was 357 minutes (303, 493) and 371 minutes (252, 424) in t-Branch and CMD procedures, respectively (*p* = .34).

The median length of stent graft covered aorta was 435 mm (397, 481) and 341 mm (284, 446) (*p* = .02), resulting in median 80% (73, 89) and 63% (52, 86) aortic coverage in t-Branch and CMD patients, respectively.

### Thirty day outcomes

Two electively operated t-Branch patients died of multi-organ failure, yielding a 30 day overall mortality of 4%, divided between 7% (*n* = 2) and 0% in t-Branch and CMD groups, respectively (*p* = .52).

SCI developed in five (18%) t-Branch (two paraplegias and three paraparesis) and two (12%) CMD patients (two paraplegias) (*p* = .69), correlated with >400 mm of stent graft aortic coverage (*p* = .05) and Crawford type II aneurysms (*p* = .01). No difference was seen between early and late patients (*p* = .414).

Re-interventions were performed on three (11%) t-Branch patients and four (24%) CMD patients, indications for all being stent corrections (*n* = 3) and thoracic stent graft implantation for a type 1a endoleak (*n* = 1) (*p* = .40).

Median days to hospital discharge was 10 (7, 19) for t-Branch patients and 8 (6, 11) for CMD patients (*p* = .12).

### Follow up

Follow up time was 26 (14, 47) and 25 (12, 52) months for t-Branch and CMD patients, respectively.

Aneurysm shrinkage occurred in nine (32%) and six (35%) t-Branch and CMD patients, respectively (*p* = 1.0), while an increase was detected in five (18%) t-Branch patients and four (23%) CMD patients (*p* = .93).

**Figure 1:**
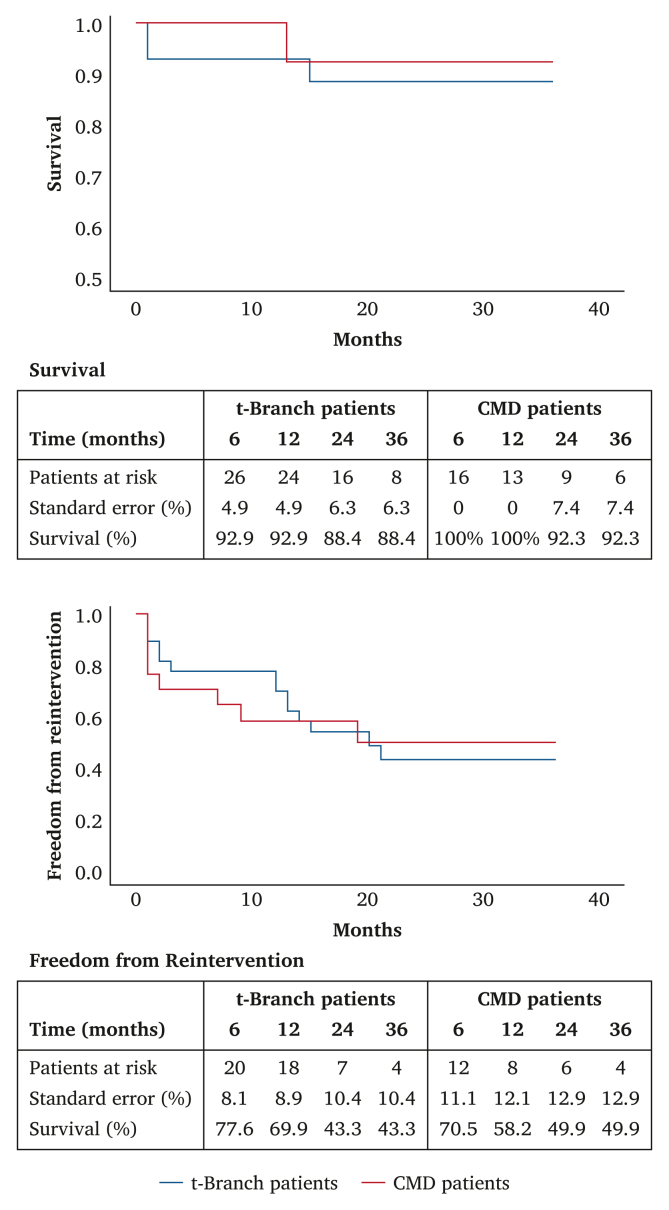
Kaplan–Meier analyses of survival and re-intervention of patients with thoraco-abdominal aortic aneurysms treated with the multibranched off the shelf Zenith t-Branch stent graft or a custom made device (CMD).

Estimated survival at one, two, and three years was 93, 88%, and 88% for t-Branch, and 100%, 92%, and 92% for CMD patients (*p* = .62) (Fig. 1). One t-Branch patient died from autopsy verified aneurysm rupture at 15 months.

SCI improved in two t-Branches during follow up, yielding a permanent paraplegia rate of 7% (*n* = 2) and 12% (*n* = 2) for t-Branch and CMD patients, respectively.

Freedom from re-intervention at one, two, and three years was 70%, 43%, and 43% for t-Branches, and 58%, 50%, and 50% for CMDs (*p* = .94), detailed in Fig. 1. Target vessel patency at the end of follow up was 94% and 96% among t-Branches and CMDs, respectively.

## Discussion

In this investigation of 45 TAAA patients treated with t-Branch or CMD, 30 day mortality was low, technical success was achieved in 87% of the procedures, and overall two year survival was 90%.

The combined 30 day mortality rate of 4% seems acceptable considering the highly comorbid patient population. Mortality rates of 7% (2/28) among t-Branch patients and 0% among CMD patients compares well with f/bEVAR results reported by other institutions, with 5.8% in meta-analysis of t-Branch studies by Konstantinou and colleagues, and with 7.8% in a 10 year summary of 166 CMD patients by Verhoeven and colleagues.^11^^,^^12^ The t-Branch material consisted of a combination of acute (*n* = 5) and elective (*n* = 23) cases, probably contributing to the somewhat lower early mortality rate experienced. The non-statistically significant difference in mortality rate between the stent graft systems aligns with results from Bisdas et al.^5^

Combined technical success was 87%. In a t-Branch meta-analysis, Konstantinou and colleagues found rates ranging from 63% in all ruptured aneurysm patients to 100% in all elective cases, pooled at 93%.^12^ As in the present study, Silingardi and colleagues described a combination of urgent and planned procedures and reported 92% technical success.^9^ All unsuccessful t-Branch cases were related to renal artery repair, with two arteries being damaged on cannulation and one suffering a type 1c endoleak. CMD performance also ranges below that of other f/bEVAR studies, where two major studies in the field report technical success in 95% and 94% of procedures, respectively.^11^^,^^16^ Eagleton and colleagues do not, however, include absence of type 1 or 3 endoleaks in their definition. Using their definition, the present authors’ technical success would be comparable at 94% (16/17).

In a publication from 2013 on t-Branch patients, Bosiers et al. reported SCI rates of 33%.^6^ Later experiences show that lower SCI rates are generally achieved, pooled at 12% in the t-Branch meta-analysis.^12^ SCI among patients receiving CMDs ranges lower, with Verhoeven and Eagleton reporting SCI at 9%.^11^^,^^16^ Further confirming this, a meta-analysis comparing endovascular and open surgery, found a pooled SCI rate of 8.8% among 17 endovascular TAAA studies.^17^ The findings, although not statistically significant, follow this tendency of SCI occurring more frequently with t-Branch patients.

This trend is not surprising, as the present authors, like others, demonstrate a significant correlation between the length of graft covered aorta and SCI.^3^ t-Branch treatment typically requires thoracic components for proximal attachment, which leads to sacrifice of several centimetres of healthy aorta in Crawford type IV aneurysms.^4^ Thus, in this study many of the nine type IV aneurysms were practically turned into type IIIs in terms of coverage. One type IV patient developed SCI, which might have been avoided with a patient specific CMD. High SCI rates were identified in preliminary results, thus from 2019 onwards CMDs were preferred in planned procedures, especially in aneurysms with shorter expected coverage lengths.

Re-interventions were performed on 49% of patients, most of which were performed early in the follow up period. Cone beam CTs were not used routinely at the end of procedures, which could explain the high early re-intervention rates, as re-intervention indications were first identified on CTA ahead of discharge.

The main limitations of this study are the small sample sizes and the retrospective design. Furthermore, there are no data on patients who were turned down for intervention because of comorbidities, and the study does not report on patients treated with open surgery in the same time frame.

### Conclusion

Low 30 day mortality and acceptable technical success and spinal cord ischaemia rates were found in patients treated with endografts for TAAAs. Medium term survival was high, and there were no statistically significant differences in clinical outcomes between patients treated with the Zenith t-Branch and CMD.

## Funding

10.13039/100009123Norwegian University of Science and Technology, Faculty of Medicine and Health Sciences.

10.13039/501100011769St. Olavs Hospital, Trondheim University Hospital.

## Conflict of interest

None.
